# Impacts of pregnancy among *quilombola* adolescents

**DOI:** 10.1590/1518-8345.6239.3843

**Published:** 2022-11-25

**Authors:** Laisa Silva Santos, Ariane Cedraz Morais, Rita da Cruz Amorim, Sinara de Lima Souza, Lucas Amaral Martins, Aisiane Cedraz Morais

**Affiliations:** 1 Universidade Estadual de Feira de Santana, Departamento de Saúde, Feira de Santana, BA, Brazil; 2 Universidade Federal do Recôncavo da Bahia, Centro de Ciências da Saúde, Santo Antonio de Jesus, BA, Brazil

**Keywords:** Adolescent, Pregnancy in Adolescence, African Continental Ancestry Group, Health Policy, Nursing, Qualitative Research

## Abstract

**Objective:**

understand the impacts of pregnancy on the lives of *quilombola* adolescents.

**Method:**

this is a qualitative study using an exploratory descriptive design. Ten *quilombola* mothers who experienced pregnancy during adolescence participated in this study. Data were collected through semi-structured interviews and then submitted to content analysis.

**Results:**

three categories were identified, showing the knowledge of the participants about being a *quilombola* and about the impacts on their lives after discovering they were pregnant and how they handled the fact of being a mother-adolescent according to the adaptations and expectations of their new phase.

**Conclusion:**

pregnancy in adolescence had impacts on several aspects of the lives of adolescents, with psychological, educational, socioeconomic, and family implications, as well as new responsibilities. Also, this study highlights the role of pregnant adolescent reception of nursing professionals to meet the individual needs of adolescents and develop prevention strategies to help reduce pregnancy among *quilombola* adolescents.

## Introduction

Adolescent pregnancy has become a serious public health problem^1^. Data from the Ministry of Health (MS) in Brazil show that more than 20,000 girls under the age of 15 years become pregnant every year^2^. In addition, several factors lead to adolescent pregnancy, including: socioeconomic aspects, low educational level, reduced age both at menarche and at first sexual intercourse, lack of information about contraceptives or programs that support adolescents^3^. Early pregnancy affects the personal-social context, causing biological, psychological, economic, and family changes, school abandonment, and financial dependence on parents^4^.

Although pregnancy rates have dropped in recent years in Brazil^5^ ^-^ ^6^, the adolescent pregnancy rate is high, with 400,000 cases every year. Regarding the age group, data show 28,244 children were born to mothers aged 10 to 14 years and 534,364 children to mothers aged between 15 and 19 years in 2014^6^, with higher prevalence among black women, with low educational and socioeconomic levels, in the North and Northeast regions^7^ ^-^ ^8^ of the country. Considering these data, social inequality and discrimination make Brazilian adolescents victims of exclusion and misery, showing a growing trend of events that increase impoverishment and consequent social vulnerability, exposing black women, from an early age, to situations that put them at physical, psychological, and social risk^9^.

Despite the national policy focused on adolescence^10^, it does not consider the specificities of different adolescences, based on aspects of ethnicity, history, and transculturality. In this context, *quilombola* communities, comprised of Afro-descendants according to self-attribution criteria and which present cultural singularities, are victims of social vulnerability, with an impact on their health conditions, especially those located in rural areas^11^. Then, nursing care is key when planning strategies for the prevention and monitoring of adolescent pregnancy, since nurses are inserted in the context of these communities and they know the real needs and vulnerabilities of this population. These strategies must include sexual and reproductive health, youth protagonism, life project, in addition to family involvement, and multidisciplinary actions.

The influence of health inequality inherent to the context of black *quilombola* adolescents is also highlighted, justifying the need to investigate this theme. Also, in a search to analyze the status of this theme in the Scientific Electronic Library Online (SciELO) and the Virtual Health Library (BVS) databases, using descriptors *quilombola, adolescents,* and *nursing*, with Boolean AND, no study was found addressing nursing care regarding the impact of pregnancy on *quilombola* adolescents.

Given the above, this study aimed to understand the impact of pregnancy on the lives of *quilombola* adolescents. Its results may support planning of nursing care focused on *quilombola* adolescents who experience early pregnancy.

## Method

### Study design

This is a field study, with an exploratory descriptive qualitative approach^12^, based on the Consolidated Criteria for Reporting Qualitative Research (COREQ)^13^.

### Study site, period, and participants

This study was conducted in the *quilombola* community of São Francisco do Paraguaçu, located in the municipality of Cachoeira, Bahia, Brazil, between October 25 and 30, 2021. Ten mothers from this community who experienced pregnancy during adolescence participated in the study. This group was selected due to the scarcity of studies addressing this theme in the *quilombola* population.

The following inclusion criteria were adopted for participant selection: *quilombola* mothers who have experienced pregnancy during adolescence, considering a maximum period of three years for short-, medium-, and long-term impacts of pregnancy. The exclusion criteria were: deaf mothers, due to the researcher’s limitation while adjusting the collection technique; and mothers of children with special needs, considering this condition implies changes in the daily routine of maternal care. No participant was excluded.

The participants were contacted after requesting the coordination of the local *quilombola* association, which allowed the selection of mothers for the study and individual contact at their homes.

Although qualitative studies do not previously restrict the number of participants, and data collection takes place until the study topic is fully understood, it was initially defined that ten mothers would be interviewed, and it was coincidently the number of interviews performed to obtain saturation of empirical data. No participant refused to participate or withdrew from the study.

### Instruments and data collection

Semi-structured interview was used as a data collection technique^14^. The instrument had two sections: section one presented sociodemographic data of participants and section two presented the guiding questions: Talk about what it means for you to live in a *quilombola* community. Talk about how the pregnancy experience was for you. Tell me what changed in your life after your first pregnancy. Tell me what changed in your life after your child was born.

Data were collected by the researcher, who is a nursing course student and lives in this community. All ethical requirements were met. To facilitate data collection, contact was made with the head of the local resident association, who issued a consent form authorizing data collection.

The interviews were conducted at the homes of participants in the morning or afternoon, according to their availability. They lasted 20 minutes on average and were recorded without interference of third parties. The interviews were later transcribed to ensure data reliability.

Identification codes were used to protect the identity of the participants and ensure confidentiality: MQ1, MQ2, … MQ10 (*quilombola* woman 1, 2, … to 10). Data collection ended when theoretical data saturation was reached, i.e., repetition of participant’s narratives.

The eligible women signed an informed consent form. Neither an assent form nor authorization from guardian was required because all mothers were over 18 years old. Then, the data collection procedure was explained, and the women were asked for permission to record their testimonies.

### Data processing and analysis

The content analysis proposed by Minayo^15^ was used with the identification of thematic categories based on the units of meaning, according to the following steps:

1. Data ordination: data obtained in the field work were mapped with the transcription of interviews, rereading of the material, and organization of the accounts, allowing a better understanding of the content.
2. Data classification: through an exhaustive and repeated reading of the texts, thematic categories were identified according to the units of meaning. A synthesis of the units of meaning was developed, leading to the definition of dimensions of analysis. Three categories were created: 1. *quilombola* identity in the perspective of women; 2. impacts of pregnancy in adolescence; and 3. discovery of maternal identity.
3. Final analysis: articulations were established between data and the theoretical frameworks of the study through discussion among the authors.

### Ethical aspects

This study observed the ethics criteria of Resolutions nº 466/2012^16^ and 510/2016^17^ ^)^ for research with human beings and was approved by the research ethics committee, approval nº 5.053.597.

## Results

Ten mothers participated in the study, aged 18 to 22 years. Regarding education, only two had a higher education degree, one had high school degree, and seven had not concluded elementary school.

In terms of occupation, most participants (eight) performed activities in common, such as fishing, cleaning shellfish, cleaning houses, and selling clothes, while two of them only studied. Race/color was defined by self-classification: eight self-declared black and two self-declared brown. Regarding their marital status, eight were single, but two of them reported having a romantic relationship (stable union) with a partner, the child’s father. Regarding the number of children, only one of the participants had two children; the others had one child each. Regarding the monthly family income, only two received up to one minimum wage while the others received lower amounts.

Based on the analysis of the interviews, the following categories were defined: *quilombola* identity in the perspective of women, impacts of pregnancy in adolescence, and discovery of maternal identity.

### *Quilombola* identity in the perspective of women

This category showed the knowledge of participants about being *quilombola* and how they characterize the ethnic identity of the community, according to the narratives below:

*So, for me, being a quilombola is fighting for our rights, it means staying strong in the fight, prioritizing our rights, customs, valuing our culture.* (MQ6)

*Living in a quilombola community means resistance in all processes of fight, of resistance that our ancestors fought for.* […] *The word itself says it all, right? Resistance, resisting the various forms of oppression, especially prejudice, racism.* (MQ1)

While talking about cultural appreciation, one of the participants highlighted:

*Today many things are lost too, right?* […] *samba circle, candomblé parties, in the past, we had capoeira circles too, street prayers. Even here today there are many evangelical churches that were not here before, my grandfather said they only had candomblé and the Catholic Church.* (MQ6)

The participants also talked about interactions in the community, their subsistence, and their rights for living in a *quilombola* community:

*It’s very calm here, we don’t have violence* [...] *the head of the association gives us the basic food baskets, to all the community, it works for many things* […] *because not everyone has good conditions here, fishing and agriculture are our subsistence.* (MQ5)

*I think that quilombola, for being a quilombola, deserves all these benefits.* […] *The head of the quilombo runs after that and brings that to us.* […] *Basic food baskets, he also brought this wood stove, which is very good, we also have a house, my house is my life, it is very good.* (MQ4)

### Impacts of pregnancy in adolescence

In this category, we will discuss the implications in the lives of these adolescents after the discovery of pregnancy, mainly related to the psychological, educational, socioeconomic, family, and social aspects.

Regarding the psychological aspects of the experience of becoming pregnant in adolescence, the participants mentioned several feelings, in addition to the desire for motherhood.

*It wasn’t a planned pregnancy, so it was a surprise that we didn’t plan.* […] *it touched me a lot psychologically, I had many plans, dreams and the beginning. The discovery was really a surprise, a shock* [...] *all these things generated a feeling of distress, I can’t say it was something good, that it was calm; it was a very difficult period, we feel sad, we reject it at first, I was sad, I cried, I said it was not possible.* (MQ1)

*I felt everything, afraid of taking care of the baby and then doing something wrong, I was also angry with myself because I made the mistake of getting pregnant early and losing all my youth.* (MQ8)

*It was good, I wanted to get pregnant.* […] *I planned it.* […] *I didn’t regret it, no, but I had a lot of difficulties, because it was all new to me.* (MQ3)

In view of the future perspective of the participants, most of them dropped out of school as a consequence of the pregnancy in adolescence, as stated by MQ3, MQ4, and MQ5. However, those who were in higher education interrupted school activities.

*I stopped studying because I couldn’t take care of my son and study.* (MQ3)

*I wouldn’t be able to study and take care of my son, I was very tired of taking care of him, I didn’t have the strength for anything, not even to study.* (MQ4)

*I didn’t know what to do, I had just finished high school and had a lot of projects in my mind.* […] *Then I was admitted to college a few months after the pregnancy and, again, I was desperate. I had to interrupt college studies and go back to the community.* (MQ5)

*I had to stop several activities that I could be performing at the university and my trajectory was difficult in this sense, there were failures. I couldn’t fully experience the university, I didn’t participate in several seminars, debates. All because of the pregnancy.* (MQ1)

In addition to the school dropout narrated by the participants, they had to start working. These jobs consisted mainly of fishing, shellfish cleaning, and other activities, such as house cleaning:

*I work, I clean houses to give him things, his father doesn’t give him anything. He receives government support, it helps too.* (MQ2)

*I catch crabs and fish, I didn’t have to do any of that before, because I had my mother and father to give me things* [...]*, but I had to start working, catch crabs so I could have money to eat.* (MQ4)

Regarding the family aspect, the participants highlighted the reaction of their relatives to the news of pregnancy:

*In the beginning it wasn’t very good, no, I had a lot of criticism. They said I was too young to get pregnant. But I also had help from the people of my family, so it was easier.* (MQ3)

*They didn’t like it, because I was too young. They said I was young, that I wasn’t old enough to have a child. But my mother also helped me on the first days after my son was born.* (MQ4)

Also regarding family support, the participants highlighted the reaction of their partners when they found out they were pregnant:

*He stayed by my side, he came to talk to my mother. It wasn’t something easy to handle because we could imagine the problems, but I had this support, both emotional and financial, because he was already working, so it was easy for me when compared to several teenagers who have no emotional or financial support.* (MQ1)

*He liked it, he wanted it, I didn’t like it very much because I take care of the baby, right? He helps me just a little. He only helps to give food, but he doesn’t help me take care of the child, then I have to do it by myself.* (MQ5)

Regarding the social aspect, social changes were reported as a result of the new phase:

*I also stopped hanging out with my friends, those things, because we have to be responsible and take care of the child.* (MQ5)

*I had to get away from my friends, all this generated a feeling of distress.* (MQ1)

### Discovery of maternal identity

In this category, the participants explained how it felt to be a mother as an adolescent, especially in relation to adaptations and expectations in the new phase:

*During pregnancy, we adjust plans, we adapt and plan, right? So I started to assume new responsibilities, I acquired more responsibilities, so, after the birth of my son, I had to learn how to be a mother, how to take care of a child, so the baby wouldn’t get sick because of the care I had to provide until he was old enough and today it’s easier.* (MQ1)

*I had to learn, I had to accept it, I couldn’t do anything. My biggest problem was that I didn’t know how to take care of a child, when it was time to breastfeed, it was really bad because I didn’t have milk.* (MQ4)

## Discussion

In *quilombola* communities, residents report feelings of ethnicity, identity empowerment, and belonging to the territory^18^, which are then interpreted as material and symbolic survival of the *quilombola* identity and its continuous reaffirmation^18^. Then, when talking about being a *quilombola*, the participants recalled the process of resistance to the different forms of oppression that black people, *quilombo* remnants, faced in the past when fighting for dignified living conditions. In agreement with the participants, many studies report *quilombo*s were the most significant manifestation of captive resistance ever seen in Brazil and became synonymous with fight for rights^19^.

The participants also pointed out the racial discrimination they have suffered and the importance of cultural appreciation, because they see situations of prejudice related to their cultural celebrations. This social exclusion is the result of a historical construction based on the enslavement and marginalization of this population, which for a long time did not have access to basic rights. Some ethnic-racial groups are still affected by discrimination and find it difficult to insert themselves into Brazilian society^20^ - as indicated by the higher rate of illiteracy, violence, hunger, difficult access to health services, among others.

The *quilombola* groups constitute an important segment of the population that has fought for their rights in society. These issues involve the paradox of recognizing their identity and specificities; recognition, ownership, and possession of their lands; preservation and appreciation of their culture; fight against racism and racial discrimination, among others^21^.

When talking about cultural appreciation, one of the participants highlighted her community has experienced a process of transculturality, in which traditional cultures no longer exist. This process happens when some groups adopt aspects from different cultures, creating a hybrid or new culture^22^, i.e., this process involves both the loss of the cultural identity of the community and the acquisition of new cultural phenomena. In this sense, health professionals, especially nurses, have an important role in rescuing popular health practices among the *quilombola* population in order to value typical transgenerational care in traditional communities.

The participants consider the experience in the *quilombola* community as something good, beneficial, because the environment does not offer risks, in their opinion. For them, being a *quilombola* means being a subject of rights as they mention the community receives several benefits through local representatives, because the population has a low socioeconomic level and fishing and agriculture are their subsistence activities.

It was also noticeable how the presence of a *quilombola* leader has a positive impact on them, as the leader’s actions allow benefits and rights for the community. However, the testimonies of the participants show non-protagonism of women, as they settle down in the context, not showing future perspectives beyond what is offered to them by governmental and/or local policy. Then, we emphasize the need for health professionals to reformulate their practices aiming to redefine the image of adolescents and promote alternative and creative practices that value youth protagonism^23^.

Regarding future projects, there is no evidence of medium- and long-term planning of participants, not to mention old plans abandoned prior to pregnancy. When comparing our findings to those of a study with pregnant adolescents^24^, the results were similar only with girls who had been submitted to violence, so we can associate the historical context of *quilombo* with the absence of life projects.

When thinking about the rights attributed to the remaining *quilombolas*, it is important to formulate public policies that can promote social equity and equal rights to this segment, since there is still an immeasurable debt inherited from the historical and social process that involves the creation of the country, whose influences have influenced its existential conditions^21^.

Regarding local participation, every *quilombo* is, in general, organized with family lands. The local political and legal organization of *quilombo*s is also highlighted, as well as the possibility of accessing government programs or financing projects with other institutions^25^.

From a political point of view, health professionals should know the government policies that support *quilombo*s and articulate with local representatives in order to develop a closer relationship with these communities.

Pregnancy among *quilombola* adolescents causes several changes in the lives of boys and girls and is related to major emotional, educational, social, and economic consequences^26^. Unplanned or unwanted pregnancy can generate different feelings in a pregnant woman^27^, such as insecurity, fear, shame, as well as loss of autonomy and higher risks of depression and suicide. Then, nurses, when providing prenatal care, must pay attention to the feelings of the adolescents during pregnancy, and refer them to specialized services of mental health care.

On the other hand, pregnancy may be desired by young women as a way to access a new status of identity and recognition through the maternal role^28^. Motherhood, in these cases, can be seen as an occupation, a role that gives a meaning to their lives. In the absence of other life projects or in case of challenges to establish alternative plans, pregnancy can be perceived by the adolescent as recognition or a path to finding her own space in the family or surrounding environments^28^.

Due to various social, political, and cultural elements, most black and poor adolescents living in Brazilian outskirts are generally unable to build their school and professional trajectory and enter the job market to ensure financial independence^29^. Then, they also end up finding a perspective of autonomy in pregnancy.

The challenges faced by these women - as pregnant adolescents - to continue their school routine, whether for financial reasons, lack of family support, or emotional conditions, show that school dropout is a critical finding to help understand that maternity in adolescence directly affects the construction of life projects. In addition, pregnancy in this age group directly impacts the perpetuation of the cycle of poverty and misery^30^, because, with the new responsibilities, adolescents drop out of school, enter the job market without training, and accept poor employment relationships.

These impacts of adolescent pregnancy, not only in *quilombola* communities, indicate the actions of health professionals, particularly nursing teams, must be based on intersectoral strategies produce to ensure positive results in health status, level of education, and quality of life of adolescents, including articulations with other sectors and professionals to help reduce school dropout^31^.

Although adolescent pregnancy is a greater obstacle for black poor young women, it constitutes a factor that changes the socioeconomic conditions of mothers, who see in higher education training a possible element of socioeconomic transformation^32^, a condition that is not present only among *quilombola* women.

The participants clearly show a concern regarding the financial condition of their parents as they had to deal with a new member in the family, associated with the instability of the family’s subsistence. For this reason, they need to work to contribute to the family income and fulfill their own needs and those of their child(ren). In this context, school dropout is a consequence of adolescent pregnancy^32^ and ends up reducing the chances of these young women to find a formal job. This fact has an impact on the socioeconomic aspect, as most participants have informal jobs, such as fishing, shellfish cleaning, and house cleaning.

Besides the feelings resulting from the discovery of pregnancy, the participants had to deal with the opinion and acceptance of pregnancy by their parents, who initially expressed criticism, dissatisfaction, and concern about the future of their daughters.

In general, the participants reported that in the beginning of pregnancy, when they told their parents about it, they did not receive the expected support^33^. But, over time, the pregnancy was accepted by the family, and they had the necessary support^33^. The mother figure was a “stronghold”, allowing the adolescent to experience the pregnancy and birth process with tranquility^34^.

Regarding the child care being attributed to the mother, our findings show changes in the behavior of the adolescent father^11^. During pregnancy, he shares the care but, later, he assigns to mothers the responsibility for the child education and care at home, and the father becomes the family provider.

According to our study, pregnancy causes changes in the social cycle of the participants, so they assume new behaviors, showing responsibility and care due to the arrival of the child, as well as distress for having to be away from friends.

First pregnancy in adolescence has an impact on the adolescent’s personal, family, social, and educational life. In most cases, pregnancy changes her school life and makes her distant from social groups and life projects^35^.

With regard to friends, social support is extremely important for the acceptance of the pregnancy, because, when feeling supported, adolescents see pregnancy as a positive event. Then, strategies should be implemented to encourage stronger connections between the young woman and her family, partner, and friends so that she feels supported^34^.

Motherhood in adolescence causes constant and intense changes, as becoming a mother is a big challenge^36^, as mentioned by the participants. The reflections made about the concepts involved in becoming a mother in this period provided a better understanding of the changes in different aspects.

The statements of adolescents indicate motherhood was built as it was experienced, because, in fact, only one of the mothers showed the desire to become pregnant. The others, because of an unexpected pregnancy, were not previously prepared for it.

The pregnancy discovery and experience of the participants involved new responsibilities in this new phase, when they were concerned about the well-being of the child and had fear regarding the newborn care. Adolescents, as they adapt to the new condition of being a mother, overcome the initial difficulties, develop and solidify the bond, love and complicity with the child, through the experience over the days, a fact that shows their relationship with the child. Therefore, the adolescent gradually builds her conception of mother, living in her own way and at her own pace to develop this child recognition, assuming her responsibilities, and starting to feel more confident about her maternal abilities^34^.

Study limitations refer to the scarcity of literature on the impacts of pregnancy on *quilombola* adolescents, which limits comparisons with other studies. However, this investigation expands the theoretical understanding of this subject and helps expand the knowledge of health professionals regarding adolescent pregnancy and development of health policies focused on adolescents, especially those in situations of higher social vulnerability. The role of nursing in the care of pregnant *quilombola* adolescents is highlighted due to the nurse closeness to the community and the possibilities that prenatal care offers.

## Conclusion

Adolescent pregnancy in a *quilombola* community triggered psychological and emotional implications; educational issues associated with the high school dropout rate, which, consequently, reduced professional opportunities; and socioeconomic problems, especially because the adolescents were already in a condition of vulnerability associated in the context of a *quilombo*. Regarding family support, the participants reported rejection at first, but later they had support from their partner and family. Regarding the social aspect, distance from friends was reported as a result of the adaptations to the new phase.

The discovery of maternal identity involves feelings of motherhood acceptance, insecurities related to child care, as well as adaptation processes that are constituted as motherhood is experienced. In view of the above, it is important to strengthen health policies for this population aiming to disseminate information about preventive and educational measures, respecting, above all, the ethnic, historical, and sociocultural aspects. Then, the role of nursing is highlighted for the development of intersectoral prevention strategies that can effectively contribute to health promotion, with interventions that focus on youth protagonism, encouraging the development of life projects and safe sexuality, as well as care for pregnant adolescents in order to meet their individual needs, observing the particularities of being black and *quilombola* women.
